# Relevance of obesity-related organ damage and metabolic syndrome classification in cardiovascular and renal risk stratification in patients with essential hypertension

**DOI:** 10.3389/fcvm.2024.1369090

**Published:** 2024-03-28

**Authors:** Luigi Petramala, Antonietta Gigante, Francesca Sarlo, Adriana Servello, Francesco Circosta, Luca Marino, Antonello Ciccarelli, Giuseppe Cavallaro, Claudio Letizia

**Affiliations:** ^1^ Department of Translational and Precision Medicine, “Sapienza” University of Rome, Rome, Italy; ^2^ UOC Chimica, Biochimica e Biologia Molecolare Clinica, Fondazione Policlinico Universitario A. Gemelli I.R.C.C.S., Rome, Italy; ^3^ Emergency Medicine Unit, Department of Emergency-Acceptance, Critical Areas and Trauma, Policlinico “Umberto I”, Rome, Italy; ^4^ Department of Clinical, Internal, Anesthesiological and Cardiovascular Sciences, “Sapienza” University of Rome, Rome, Italy; ^5^ Department of Mechanical and Aerospace Engineering, “Sapienza” University of Rome, Rome, Italy; ^6^ Department of Medico-Surgical Sciences and Biotechnologies, “Sapienza” University of Rome, Rome, Italy; ^7^ General Surgery Unit, ICOT Hospital, Latina, Italy

**Keywords:** hypertension, metabolic syndrome, cardiovascular risk, organ target damage, chronic kidney disease, obesity

## Abstract

**Introduction:**

Hypertension is a relevant cardiovascular comorbidity. Adipose tissue represents a metabolically active tissue involved in the regulation of blood pressure and metabolic alterations. In recent decades, several classifications for the metabolic syndrome (MS) have been proposed. Recently, a new syndrome called the “Cardiovascular-kidney-metabolic” (CKM) syndrome was identified, to determine patients at high cardiovascular and metabolic risk. The aim of the study was to compare different classifications in a large population of hypertensive patients.

**Materials and methods:**

Between September 2022 and August 2023, we consecutively enrolled 772 hypertensive patients (407 men; 365 women; mean age 52.2 ± 15.1 years), evaluating anthropometric, biochemical, and instrumental parameters (transthoracic echocardiogram, carotid echo-Doppler, 24-h ambulatory blood pressure monitoring, fundus oculi).

**Results:**

Using different classifications we found MS prevalence: Adult Treatment Panel III (ATP-III) 28.8%, International Diabetes Federation (IDF) 31.5%, CKM 40.7%. CKM Classes 3 and 4 showed higher body mass index and waist circumference compared with other groups. Compared with ATP-III and IDF, CKM Class 4 showed higher 24-h systolic blood pressure, lower percentage of controlled hypertension, increased interventricular septum and posterior wall, reduced ejection fraction, and greater prevalence of hypertensive arterial retinal damage.

**Discussion:**

Visceral obesity and MS are frequent conditions with healthy impact, becoming an important trigger for the development of cardiovascular and metabolic complications. The different MS classifications allow the early identification of patients at high risk of cardiometabolic complications. The new CKM syndrome proves useful to identify individuals at high risk for CKM morbidity and mortality.

## 1 Introduction

Hypertension is the most prevalent cardiovascular (CV) comorbidity, affecting more than 1.2 billion adults aged 30–79 years (1). Several studies showed a direct correlation between the imbalance of CV regulatory systems and the adipose tissue, this latter representing the hemodynamic hallmark for high blood pressure (BP) and abnormalities in microcirculation (1, 2). The relationship between obesity and hypertension has been established since the Framingham Heart Study (3) and it is well known that excess adiposity increases the risk for cardiometabolic diseases, especially considering the visceral adipose tissue (4).

Obesity is characterized by increased expression of adipose tissue, with greater representation and distribution at visceral sites; adipose tissue is typically infiltrated by several cells such as white adipocytes, inflammatory immune cells, and stem cells (5); it represents a metabolically active tissue capable of secreting several adipokines involved in the regulation of physiological processes, including BP and vascular tone (6). As regards this, in a cohort of patients with comorbidities such as hypertension and diabetes mellitus admitted in a hospital for elective coronary angiography, Kannel found that abdominal visceral fat was increased in patients with concentric hypertrophy (3). Thus, the assessment of abdominal visceral fat allows one to identify, among patients with cardiometabolic comorbidities such as arterial hypertension, a leading cause of increased mortality (4).

To integrate cardiovascular and metabolic diseases in association with morbidity and mortality, several classifications have been proposed by international health organizations in an attempt to suggest a unique definition of metabolic syndrome (MS). The three most popular definitions have been recommended by the World Health Organization (WHO) in 1998, the National Cholesterol Education Program Adult Treatment Panel III (NCEP/ATP-III) in 2005, and the International Diabetes Federation (IDF) in 2006, including glucose level/insulin resistance, high BP, high triglyceride level, reduced high-density lipoprotein cholesterol, and waist circumference (WC) (7–9). Over the past few decades, the several classifications of MS were focused on the relevance of the amount of visceral fat compared with body mass index (BMI), representative of global body adiposity.

Furthermore, in the past few decades, different research groups have evaluated the reproducibility and importance of using the classifications in different populations. In Caucasian patients with diabetes, Monami et al. evaluated the prevalence of MS and the increased cardiovascular morbidity and mortality through the prognostic value of IDF criteria vs. ATP-III criteria. These authors have highlighted that IDF classification identifies a greater amount of MS patients, and significantly higher mortality rate in patients identified through ATP-III vs. IDF criteria [odds ratio (OR) 2.38 vs. 1.65] (10). Similarly, Haverinen et al. compared different MS definitions in the Finnish adult population, highlighting how these classifications induce different results (prevalence from 17.7% up to 43%) (11). Likewise, in a Middle East population MS prevalence ranged from 29% to 31.5% using different criteria, highlighting that IDF criteria better predicted pre-diabetes and diabetes (OR 3.4 and 6.4, respectively), while ATP-III criteria better predicted high cardiovascular disease (CVD) risk scores (OR 13.6) (12).

Recently, a new syndrome called “Cardiovascular-kidney-metabolic” (CKM) syndrome has been defined as a disease burden related to connections among obesity, diabetes, hypertension, and chronic kidney disease (CKD) (13). The items required in CKM syndrome concern subclinical or clinical CVD, as well as anthropometric measurements and biochemical parameters. CKM classification provides a broad spectrum, differing for the type of prevention and treatment; stage 0 (no CKM risk factors); stage 1 (increased adiposity); stage 2 (addition of metabolic risk factors such as hypertriglyceridemia, hypertension, diabetes, metabolic syndrome, moderate/high risk for chronic kidney disease); stage 3 (subclinical CVD); and stage 4 (clinically present CVD) (14). As regards this, CKD is not just a relevant pathological condition, but a significant marker of organ damage, particularly if associated with altered ultrasonographic examination and the presence of altered albuminuria (15). In addition to arterial hypertension and diabetes, obesity and MS are closely related to the development of CKD, as well as to the worse progression of established CKD (16).

With this background the aim of the study was to compare prevalence of MS evaluated through the different classifications (mainly CKM, ATP-III, and IDF) in patients with essential hypertension, focusing on differences on pressure profile and subclinical organ damage in different groups. In Table 1 we have reported the parameters comparing the three classification criteria (ATP-III, IDF, CKM).

**Table 1:** Metabolic and cardiovascular-kidney-metabolic syndrome definitions.

ATP-III	IDF	CKM
Three or more of the following: • WC ≥102/88 cm (M/F) • Triglycerides >1.7 mmol/L • HDL-C <1.0/1.3 mmol/L (M/F) • BP ≥130/85 mmHg or anti-hypertensive medication • Fasting plasma glucose ≥6.1 mmol/L	Central obesity plus two or more of the following: • Triglycerides >1.7 mmol/L • HDL-C <1.03/1.29 mmol/L (M/F) • BP ≥130/85 mmHg or antihypertensive medication • Fasting plasma glucose ≥5.6 mmol/L or diabetes (DM2)	• Stage 0: no risk factors • Stage 1: excess of adiposity • Stage 2: metabolic risk factors (higher WC, arterial hypertension, hyperglycemia, hypertriglyceridemia, reduced HDL-C) or Chronic kidney disease • Stage 3: subclinical cardiovascular disease • Stage 4: clinical cardiovascular disease

WC, waist circumference; BP, blood pressure; BMI, body mass index; HDL-C, high-density lipoprotein cholesterol; DM2, diabetes type 2.

## 2 Materials and methods

### 2.1 Study design

Between 1 September 2022 and 30 August 2023, we consecutively enrolled 772 patients affected by essential hypertension (407 men; 365 women; mean age 52.2 ± 15.1 years), evaluated at the Departmental Unit of Arterial Hypertension of Policlinico Umberto I Hospital of Rome. Anthropometric measurements, fasting venous blood samples, 24-h urine collection, carotid intima-media thickness (cIMT), 24-h ambulatory blood pressure monitoring (ABPM), transthoracic echocardiography, and fundus oculi exams were conducted on all participants. Arterial hypertension was defined as BP of 140/90 mmHg or higher in three consecutive measurements or in cases where antihypertensive therapy was being taken. Secondary causes of hypertension were ruled out through comprehensive evaluation based on clinical, laboratory, hormonal, and imaging examinations, adhering to international guidelines (16).

As regards this, to avoid hormonal evaluation, in patients in whom anti-hypertensive treatment could not be withdrawn for ethical reasons, calcium-channel blockers (verapamil) or a-receptor blockers (doxazosine) were allowed at minimal doses required to achieve BP control. All patients were given a written indication on the daily intakes to be consumed regarding sodium (150 mEq/day) and potassium (50–75 mEq/day). After maintaining a regular sodium–potassium diet for 2 weeks and discontinuing interfering medications, fasting blood samples for plasma aldosterone concentration (PAC) and plasma renin activity (PRA) were collected from all participants.

The participants comfortably lay in a clinostatic position for at least half an hour before venous sampling, which was performed in the morning (between 8:00 and 9:00 a.m.). A cutoff upright PAC/PRA ratio of more than 30, in the presence of aldosterone higher than 15 ng/dl and suppressed PRA, served as a screening test for primary aldosteronism. Patients with a PAC/PRA ratio exceeding 30 underwent an isotonic saline infusion (0.9% NaCl at 500 ml/h for 4 h) as a confirmatory/exclusion test. Those whose PAC decreased to less than 5 ng/dl after saline infusion were definitively excluded from having primary aldosteronism. Individuals with clinical history or clinical symptoms or electrocardiogram, echocardiographic, and angiographic signs of valvular or pericardial diseases were excluded. Individuals with hepatic diseases or with drug abuse histories were excluded, as well as those patients taking systemic corticosteroids. Renal disease was defined as reduced glomerular filtration rate (GFR): <60 ml/min/1.73 m^2^. Chronic liver disease was defined with clinical history, signs, symptoms, or laboratory findings of hepatitis, cirrhosis, or liver failure. Patients had a stable body weight for the 6 months before the study. This study adhered to the guidelines of the Declaration of Helsinki; clinical data were obtained from routine clinical practice, so we have requested consent from the Local Ethical Committee of the Department of Translational and Precision Medicine. The study design was clearly explained in layperson language and provided to each participant, who provided written informed consent.

### 2.2 Anthropometric measurements

Anthropometrics were conducted on all participants. Standing height was measured on barefoot to the nearest 0.5 cm. Weight was measured in light clothing with a platform scale to the nearest 200 g. The scale was standardized to 0 before each use. Waist circumference was taken to the nearest 0.1 cm with a standard tape measure over the abdomen at the smallest diameter between the costal margin and iliac crest. Hip circumference was measured to the nearest 0.1 cm with a non-stretchable standard tape measure. Measurements were taken over light clothing at the level of the greater trochanter (usually the widest diameter around the buttocks). For both waist and hip measurements, the tape measure was kept horizontal and just tight enough to allow the little finger to be inserted just under the tape. Two measurements were taken, and the average was used for this analysis.

### 2.3 Metabolic syndrome classification

The patients were divided into six groups according to the following definitions. ATP-III; IDF; CKM 1: Stage 1; CKM 2: Stage 2; CKM 3: Stage 3; and CKM 4: Stage 4 (15).

### 2.4 Ambulatory blood pressure monitoring

ABPM was conducted using the oscillometric technique, employing a portable, lightweight, non-invasive monitor equipped with a self-insufflating cuff (Spacelabs Medical, 90207, Issequah, WA, USA). ABPM readings were taken at 15-min intervals from 6 a.m. to midnight and at 30-min intervals from midnight to 6 a.m. Various parameters were assessed, including average daytime systolic blood pressure (SBP), average daytime diastolic blood pressure (DBP), and average daytime heart rate, as well as average nighttime SBP, average nighttime DBP, and average nighttime heart rate. In addition, the average 24-h SBP, average 24-h DBP, and average 24-h heart rate were calculated. Subject diaries were used to determine periods. The term “dipper” was applied when there was a nighttime SBP and DBP reduction of more than 10%. Ambulatory hypertension was defined as 24-h BP exceeding 130/80 mmHg (17).

### 2.5 Transthoracic echocardiography

Transthoracic 2D-guided M-mode echocardiography was executed by an expert cardiologist using commercially available equipment (Esaote MyLab Omega); with the following measurements: left ventricular end-diastolic diameter (LVEDD), left atrial diameter (LAD), wave A/E ratio, interventricular septum (IVS), left ventricular posterior wall (PW) thicknesses, aortic diameter (Ao), and ejection fraction (EF) evaluated by the Simpson method; left ventricular mass (LVM) and LVM indexed for body surface area (LVMi) were calculated according to the American Society of Echocardiography (ASE) guidelines (18).

### 2.6 Measurement of carotid intima-media thickness

A Hewlett-Packard Sonor 5,500 Ultrasound system (Hewlett Packard, Andover, MA, USA), equipped with a 3.11 MHz real-time B-mode scanner, was utilized for the assessment. Imaging of the right common carotid artery (CCA) was conducted with the individuals turning their head 45° to the left. High-resolution images were analyzed to determine cIMT, defined as thickness of the vascular intima-media complex measured in five consecutive regions of the CCA wall, with measurements taken every 4–5 mm starting near the bifurcation. The average cIMT value for each individual was calculated based on measurements from five points on both the left and right carotid arteries. Intra- and inter-observer variabilities for cIMT were 4.6 ± 0.4 and 5.2 ± 0.3, respectively. The mean common carotid diameter was determined as the line representing the media-adventitia interface from near to far wall, automatically calculated by averaging measurements at 0.1 cm intervals over 1 cm.

### 2.7 Fundus Oculi exam

All subjects underwent a bilateral funduscopic examination through an ophthalmoscope inspection. The cup-to-disk ratio, the presence of cotton wool spots and/or flame hemorrhages, and artero-venous crossing points were evaluated. Retinal damage related to microangiopathy was evaluated by an expert ophthalmologist, and organ damage was considered consistent with retinopathy greater than or equal to a second stage.

### 2.8 Chronic renal disease

Renal function was calculated with the estimate glomerular filtration rate (eGFR) equation/algorithm according to the largely used formula, defined as the Chronic Kidney Disease Epidemiology Collaboration (CKD-EPI) equation, expressed as: 141 min (sCr/k, 1)^Age^ × max (sCr/k, 1) − 1.209 × 0.993^Age ^× 1.018 (if female)_1.159 (if black), where k is 0.7 for females and 0.9 for males, a is −0.329 for females and −0.411 for males, min indicates the minimum of sCr/k or 1 and max indicates the maximum of sCr/k or 1 (19). Compared with other classifications, the CKD-EPI equation was chosen as a better assessment of renal function for normal and mild reduced eGFR >60 ml/min/1.73m^2^, mostly represented in our patient population. The presence and stage of CKD were characterized according to the Kidney Disease Outcomes Quality Initiatives (K-DOQI) guidelines as follows: stage 1 (>90 ml/min), stage 2 (60–89 ml/min), stage 3A (45–59 ml/min), stage 3B (30–44 ml/min), stage 4 (15–29 ml/min), and stage 5 (<15 ml/min) (20).

### 2.9 Statistical analysis

All data are expressed as mean standard deviation (SD). Differences between means were assessed by Student's t-test or the Mann–Whitney *U* test in non-normally distributed data for two-sample comparison, or by one-way analysis of variance (ANOVA) applying the Fisher least significant difference *post-hoc test* for multiple comparisons. Chi^2^ statistics were used to assess differences between categorical variables. Statistical significance was considered for *p*-values less than 0.05. Data analysis was conducted using dedicated statistical software SPSS (Statistical Package for Social Sciences software, version 24; SPSS Inc., Chicago, IL, USA) and e GraphPad (version 5.0; GraphPad Software, Inc., La Jolla, CA, USA).

## 3 Results

**Figure 1:**
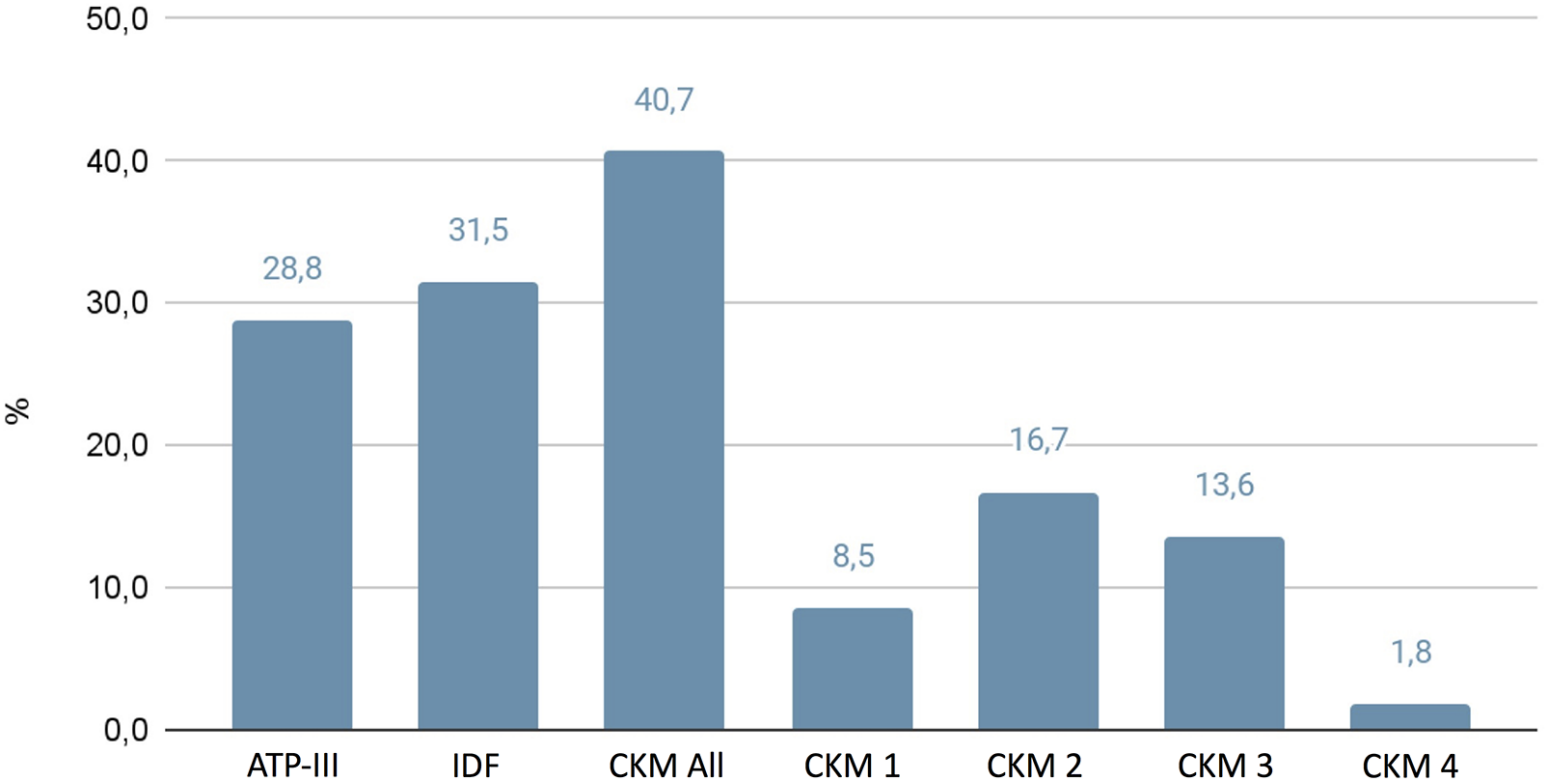
Prevalence of metabolic syndrome evaluated through different classifications.

A total of 772 essential hypertensive patients (407 men; 365 women; mean age 52.2 ± 15.1) were included in the study. In Figure 1 we have reported the prevalence of MS based on the different classifications: ATP-III 28.8%, IDF 31.5%, CKM 40.7%. The anthropometric and biochemical characteristics of the examined patients with hypertension are reported in Tables 2, 3. No differences were assessed between the several groups regarding family history of cardiovascular and metabolic pathologies. Overall, the individuals of the present study cohort were classified into six groups according to different MS classifications (ATP-III, IDF, CKM 1-2-3-4). Interestingly, the patients of CKM 3 and 4 showed higher BMI (31.4 ± 4.6 and 31.8 ± 5.6 kg/m^2^) and waist circumference (108.9 ± 10.2 cm and 118.7 ± 21.2 cm) with respect to the ATP-III groups (ATP-III 29.5 ± 5.1 kg/m^2^ and 104.3 ± 13.7 cm, respectively; *p* < 0.05).

**Table 2:** Demographic and anthropometric data of the study groups.

	Age (years)	Gender (M %)	BMI (kg/m^2^)	WC (cm)	NC (cm)	SBP (mmHg)	DBP (mmHg)	HR (bpm)
ATP-III (n.222)	55.8 ± 13.9	50	29.5 ± 5.1	104.3 ± 13.7	40.2 ± 5.1	148.1 ± 24.9	89.7 ± 13.3	71.2 ± 13.5
IDF (n.247)	55.8 ± 13.3	47.9	30.4 ± 5.4	107.4 ± 13.4	41.1 ± 4.7	150.1 ± 28.3	91.2 ± 14.1	71.5 ± 12.6
CKM 1 (n.66)	49.1 ± 15.6*	41.8	30.1 ± 3.7	105.6 ± 10.5	40.9 ± 4.2	141.1 ± 18.5	88.6 ± 13.9	72.6 ± 10.6
CKM 2 (n.129)	52.6 ± 14.8	42.6	30.1 ± 3.9	106.8 ± 11.7	40.8 ± 4.6	143.5 ± 20.2	88.8 ± 12.1	71.3 ± 12.8
CKM 3 (n.105)	58.6 ± 12.3	57.4	31.4 ± 4.6***	108.9 ± 10.2***	41.1 ± 4.7	147.4 ± 26.5	91.5 ± 14.2	70.4 ± 12.5
CKM 4 (n.14)	49.5 ± 10.3	55	31.8 ± 5.6***	118.7 ± 21.2***	42.4 ± 6.1	148.7 ± 28.4	91.2 ± 16.5	74.0 ± 4.9
*p*-value	ns	ns	***p < 0.05 vs. ATP-III	***p < 0.05 vs. ATP-III	ns	ns	ns	ns

BMI, body mass index; WC, waist circumference; NC, neck circumference; SBP, systolic blood pressure, DBP, diastolic blood pressure, HR, heart rate.

***Groups with statistically significant difference.

**Table 3:** Biochemical parameters of the study groups.

	Creatinine (mg/dl)	Glycaemia (mg/dl)	LDL (mg/dl)	HDL (mg/dl)	Triglycerides (mg/dl)	Uric acid (mg/dl)	PCR (mcg/L)	µ-Albuminuria (g/24 h)
ATP-III (n.222)	0.96 ± 0.33	105.9 ± 23.70	106.3 ± 33.9	47.6 ± 15.6	132.8 ± 92.3	5.8 ± 1.3	4,049 ± 7,608	50.7 ± 11.5
IDF (n.247)	1.03 ± 0.41	115.6 ± 34.01	105.6 ± 34.2	42.4 ± 11.7°°	142.4 ± 51.5	6.0 ± 1.5	4,939 ± 9,846	80.8 ± 78.9
CKM 1 (n.66)	0.83 ± 0.17	91.5 ± 8.9*	111.8 ± 30.2	51.5 ± 12.1	87.9 ± 27.3	5.6 ± 1.4	3,322 ± 3,564	13.7 ± 11
CKM 2 (n.129)	0.93 ± 0.19	96.5 ± 16.9	108.2 ± 33.6	50.6 ± 16.4	109.8 ± 56.8	5.8 ± 1.3	3,928 ± 6,671	39.9 ± 15.6
CKM 3 (n.105)	0.97 ± 0.29	104-9 ± 27.9	112.0 ± 39.7	51.2 ± 18.3	120.6 ± 40.8	5.8 ± 1.4	4,428 ± 6,259	36.4 ± 10.9
CKM 4 (n.14)	2.51 ± 1.13****	122.5 ± 24.8	113.5 ± 33.8	47.0 ± 9.80	117.0 ± 72.5	7.5 ± 1.4	7,550 ± 9,960	996 ± 105****
*p*-value	****p < 0.05 vs. ATP-III, IDF, CKM 1,2,3	*p < 0.05 vs. ATP-III, IDF	ns	°°*p* < 0.05 vs. CKM 1,2,3	ns	ns	ns	****p < 0.05 vs. ATP-III, IDF, CKM 1,2,3

LDL, LDL-cholesterol; HDL, HDL-cholesterol; PCR, C-reactive protein.

*^,^***^,^°°Groups with statistically significant difference.

Regarding the biochemical parameters, we found in CKM 1 lower plasma glucose behavior (91.5 ± 8.9 mg/dl) compared with the ATP-III (105.9 ± 23.70 mg/dl) and IDF (115.6 ± 34.01 mg/dl) (*p* < 0.05) groups. Moreover, the IDF group showed lower levels of high-density lipoprotein (HDL) (42.4 ± 11.7 mg/dl) compared with CKM 1 (51.5 ± 12.1 mg/dl), CKM 2 (50.6 ± 16.4 mg/dl), and CKM 3 (51.2 ± 18.3 mg/dl) (*p* < 0.05).

As expected, in CKM 4 we found significantly higher creatinine values (2.51 ± 1.13 mg/dl) compared with the other groups (ATP-III 0.96 ± 0.33 mg/dl, IDF 1.03 ± 0.41 mg/dl, CKM 1 0.83 ± 0.17 mg/dl, CKM 2 0.93 ± 0.19 mg/dl, CKM 3 0.97 ± 0.29 mg/dl; *p* < 0.05). As regards this, patients classified under CKM 4 showed higher levels of 24-h urinary excretion (996 ± 105 g/24 h) compared with those in the ATP-III (50.7 ± 11.5 g/24 h), IDF (80.8 ± 78.9 g/24 h), CKM 1 (13.7 ± 11 g/24 h), CKM 2 (39.9 ± 15.6 g/24 h), and CKM 3 (36.4 ± 10.9 g/24 h) (*p* < 0.05) groups.

**Figure 2:**
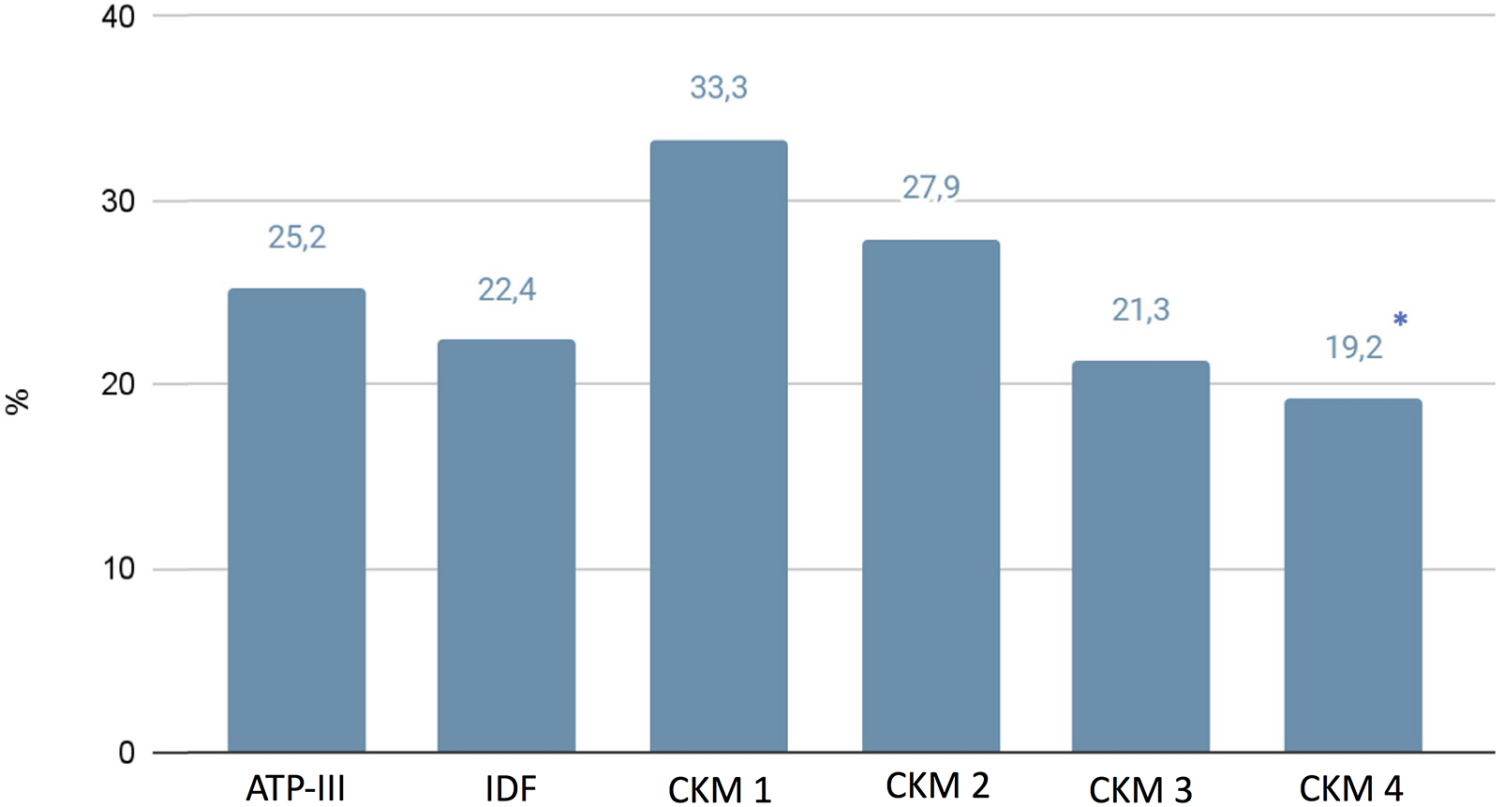
Prevalence of controlled blood pressure in studied groups.

While we did not highlight any differences regarding blood pressure behaviors clinically evaluated, using the 24-h blood pressure monitoring (ABPM), in the CKM 4 group we found significantly higher values of global 24-hr systolic blood pressure (134.75 ± 21.7 mmHg), compared with the ATP-III (130.8 ± 16.9 mmHg), IDF (132.4 ± 20.7 mmHg), CKM 1 (129.6 ± 13.3 mmHg), CKM 2 (129.5 ± 14.3 mmHg), and CKM 3 (130.9 ± 4.2 mmHg) (*p* < 0.05) (Table 4) groups. Moreover, we have found lower percentage of controlled hypertension in patients classified in CKM 4 with respect to those in CKM 1 (19.2% vs. 33.3%; *p* < 0.05) (Figure 2).

**Table 4:** Parameters obtained by ABPM and anti-hypertensive medications in the study groups.

	SBP-24h (mmHg)	DBP-24 (mmHg)	HR-24h (bpm)	SBP-D (mmHg)	DBP-D (mmHg)	HR-D (bpm)	SBP-N (mmHg)	DBP-N (mmHg)	HR-N (bpm)	Non-Dipper Pattern (%)	Anti-hypertensive medications (%)
ATP-III (n.222)	130.8 ± 16.9	78.4 ± 11.9	75.2 ± 1.8	133.7 ± 17.8	81.5 ± 11.6	77.5 ± 13.1	123.7 ± 30.7	71.4 ± 11.3	60.9 ± 11.1	28.3	2.1 ± 1.1
IDF (n.247)	132.4 ± 20.7	80.3 ± 13.9	75.0 ± 10.0	137.2 ± 17.9	83.5 ± 11.9	77.9 ± 12.5	126.6 ± 21.7	73.8 ± 12.3	70.2 ± 10.0	35.9	2.3 ± 1.2
CKM 1 (n.66)	129.6 ± 13.3	78.6 ± 10.5	76.9 ± 10.7	133.2 ± 14.3	81.8 ± 11.1	80.2 ± 11.2	120.5 ± 13.3	70.7 ± 9.7	71.0 ± 9.8	30.4	1.8 ± 1.2
CKM 2 (n.129)	129.5 ± 14.3	78.7 ± 9.9	74.8 ± 10.9	134.0 ± 4.9	84.9 ± 10.2	77.8 ± 12.9	121.9 ± 18.7	71.6 ± 10.6	68.5 ± 10.8	31.6	2.1 ± 1.1
CKM 3 (n.105)	130.9 ± 4.2	78.7 ± 9.4	75.3 ± 11.1	134.6 ± 15.9	81.8 ± 9.9	77.0 ± 11.3	123.5 ± 16.8	71.0 ± 10.3	68.5 ± 12.4	39.8	2.3 ± 1.2
CKM 4 (n.14)	134.75 ± 21.7****	93.2 ± 45.4	77.2 ± 14.2	137.0 ± 19.9	87.5 ± 14.8	71.5 ± 16.6	129.5 ± 16.7	66.5 ± 2.1	63.5 ± 0.7	38.6	2.2 ± 1.3
*p*-value	****p < 0.05 vs. TP-III, IDF, CKM 1,2,3	ns	ns	ns	ns	ns	ns	ns	ns	ns	ns

SBP-24h, 24 h systolic blood pressure; DBP-24h, 24-h diastolic blood pressure; HR-24h, 24-h heart rate; SBP-D, diurnal systolic blood pressure; DBP-D, diurnal diastolic blood pressure; HR-D, diurnal heart rate; SBP-N, nocturnal systolic blood pressure; DBP-N, nocturnal diastolic blood pressure; HR-N, nocturnal heart rate.

****Group with statistically significant difference.

Regarding the echocardiographic parameters (Table 5), patients in CKM 4 showed significant increase in the interventricular septum (12 ± 2.1 mm) and posterior wall (15.3 ± 8 mm) compared with the groups classified as ATP-III (11.3 ± 4.3 mm and 9.7 ± 2.4 mm), IDF (11.9 ± 5.8 mm and 10.0 ± 2.2 mm), CKM 1 (10.8 ± 1.7 mm and 9.9 ± 3.1 mm), CKM 2 (10.8 ± 2.0 mm and 9.7 ± 1.7 mm), and CKM 3 (11.4 ± 1.9 mm and 9.8 ± 1.4 mm) (*p* < 0.05). Interestingly, patients in CKM 4 showed a significant reduction in ejection fraction (48 ± 13.1%) compared with those in the other groups (ATP-III 56.6 ± 3.5%, IDF 56.3 ± 3.8%, CKM 1 57.3 ± 3.1%, CKM 2 59.3 ± 1.3%, and CKM 3 54.9 ± 1.2%, respectively; *p* < 0.05). Finally, the CKM 4 group showed a mean increase in the aortic bulb diameter (36 ± 10 mm), compared with the IDF group (32.4 ± 4.9 mm; *p* < 0.05).

**Table 5:** Echocardiographic parameters, prevalence of chronic kidney disease and cardiovascular organ damage in study groups.

	LVEDD (mm)	Ao (mm)	LA Diam (mm)	IVS (mm)	PW (mm)	EF (%)	IMT (mm)	FO (%)	Athero (%)	CKD-EPI ml/min
ATP-III (n.222)	47.8 ± 8.9	32.4 ± 4.9	22.6 ± 7.7	11.3 ± 4.3	9.7 ± 2.4	56.6 ± 3.5	0.83 ± 0.15	30.2	27.1	82.7 ± 22.5
IDF (n.247)	48.1 ± 4.2	33.5 ± 535	23.4 ± 7.6	11.9 ± 5.8	10.0 ± 2.2	56.3 ± 3.8	0.84 ± 0.14	32.8	24.3	80.2 ± 21.3
CKM 1 (n.66)	47.6 ± 3.8	32.6 ± 4.2	21.5 ± 7.9	10.8 ± 1.7	9.9 ± 3.1	57.3 ± 3.1	0.79 ± 0.14	21.7	24.2	100.9 ± 6.3
CKM 2 (n.129)	48.1 ± 3.8	33.4 ± 4.9	22.4 ± 8.1	10.8 ± 2.0	9.7 ± 1.7	59.3 ± 1.3	0.80 ± 0.13	31.6	16.4	85.5 ± 16.9
CKM 3 (n.105)	47.2 ± 4.4	32.7 ± 4.8	23.3 ± 6.1	11.4 ± 1.9	9.8 ± 1.4	54.9 ± 1.2	0.83 ± 0.14	37.8***	26.1	78.5 ± 23
CKM 4 (n.14)	51.0 ± 1.4	36 ± 10****	29.8 ± 9.8	12 ± 2.1****	15.3 ± 8****	48 ± 13.1****	0.85 ± 0.24	47.3***	25	38.8 ± 34****
*p*-value	ns	****p < 0.05 vs. IDF	ns	****p < 0.05 vs. ATP-III, IDF, CKM 1-2-3	****p < 0.05 vs. ATP-III, IDF, CKM 1-2-3	****p < 0.05 vs. ATP-III, IDF, CKM 1,2,3	ns	****p < 0.05 vs. ATP-III, IDF, CKM 1,2	ns	****p < 0.05 vs. TP-III, IDF, CKM 1,2,3

LVEDD, left ventricular end-diastolic diameter; Ao, aortic diameter; LA Diam, left atrium diameter; IVS, interventricular septum; PW, posterior wall; EF, ejection fraction; IMT, intima-media thickness; FO, altered fundus oculi findings; Athero, atherosclerotic plaque; CKD-EPI, Chronic Kidney Disease Epidemiology Collaboration.

****Group with statistically significant difference.

Regarding the organ damage observed at retinal microcirculation, we have highlighted a greater prevalence of hypertensive arterial retinal damage in the CKM 3 and 4 (37.8% and 47.3%, respectively) compared with the ATP-III groups (30.2%), IDF (32.8%), CKM 1 (21.7%), and CKM 2 (31.6%) (*p* < 0.05) groups. No differences were found regarding the prevalence of atheromatous plaques and intima-media thickness in the carotid arteries in the different groups.

**Figure 3:**
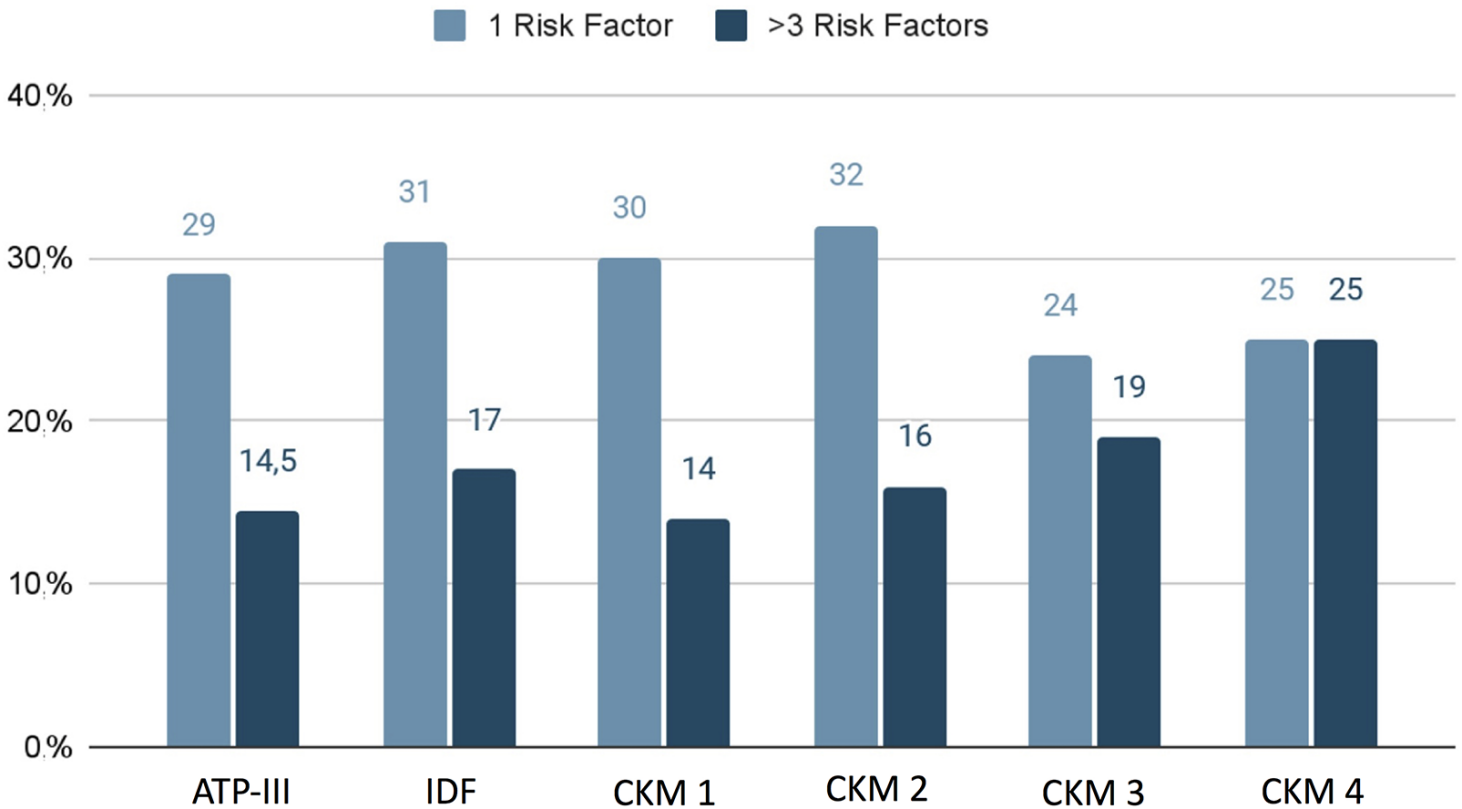
Prevalence of cardiovascular risk factors in the studied groups.

Finally, we found that in the groups classified through ATP-III, IDF, and CKM 1 and 2, we more frequently found one cardiovascular risk factor, while in the CKM 3 and 4 we observed a greater prevalence of multiple comorbidities (more than three cardiovascular risk factors) (Figure 3).

## 4 Discussion

For more than two decades the MS, an association of relevant cardiovascular and metabolic risk factors (such as visceral obesity, dyslipidemia, hyperglycemia, and hypertension), has become one of the major public health challenges (21). From its first identification called “syndrome X” by Reaven in 1988 (22), the pivotal relevance of MS definition was the identification of those subjects highly exposed to the development of both type 2 diabetes and cardiovascular disease (CVD). Over the years, different classifications of MS have been suggested; the first attempts were made by WHO and the European Diabetes groups in 1999, using insulin resistance and alterations in glucose metabolism as essential elements. Later, the US National Cholesterol Education Program: Adult Treatment Panel III in 2001 simplified the diagnostic criteria by focusing attention on cardiovascular disease risk (23), albeit not assessing ethnic differences (24). Thus, in 2004 the IDF put visceral obesity at the center of attention as an indicator of insulin resistance, differentiating the cut-offs by gender and ethnicity (8).

Recently, CKM has been proposed as a new association of metabolic and CV risk factors dependent on obesity, strictly associated to very high risk of significant complications as heart failure (HF), atrial fibrillation, coronary heart disease, stroke, and peripheral artery disease, as well as CKD (13). It is well described that excess visceral adipose tissue induces chronic inflammation, insulin resistance, and the development of metabolic risk factors for CVD and CKD, with a strong bidirectional link between dysfunction of the cardiovascular system, especially the heart, and the kidneys (25).

In our study, we found a significant prevalence of MS using the different classifications, with a higher percentage regarding the CKM classification than the ATP-III and IDF classifications. Moreover, we have highlighted that the prevalence of an increased BMI and WC is more evident in the advanced stages of the CKM classification compared with the ATP-III or IDF classifications. In these groups of patients at higher CVD risk, through ABPM we have observed greater blood pressure burden, as well as more advanced organ damage, assessed by increased 24-h urinary excretion of albumin, cardiac remodeling, reduced ejection fraction, and more advanced microangiopathy.

Recently, the review of the data obtained from the National Health and Nutrition Examination Survey (NHANES) 2011–2018 highlighted in adult obese subjects an increased risk of developing hypertension (OR 2.57–3.23) (26). In obesity-related hypertension, there is clear evidence of an imbalance of the systems regulating blood pressure behavior and chronic low-grade inflammation, favoring decreased insulin sensitivity (27, 28). Moreover, the obesity is characterized by higher oxidative stress behavior, contributing to the development of cardiovascular and metabolic comorbidities; in particular, several studies have found the activation of the main physiological source of reactive oxygen species production, NADPH oxidase (in particular the NOX2 catalytic subunit), favoring the alteration of nitric oxide synthase (NOS) activity and consequent endothelial dysfunction and cardiovascular organ damage (29). Chronic inflammation induces inadequate perfusion with consequent local hypoxia, mainly mediated by HIF-1α (hypoxia-inducible factor alpha), which represents a link with an increased production of inflammatory cytokines (30). Previously, we have shown an altered expression of different adipocytokines (leptin, adiponectin, resistin) both at circulating level and increased tissue expression in pathologies characterized by an increased cardiovascular risk (i.e., Primary Aldosteronism and Subclinical Cushing's Syndrome) (31, 32). Similarly, we have observed an increased oxidative stress in patients with hypertension with excessive aldosterone production, characterized by increased serum levels of Nox2 and urinary excretion of isoprostanes (33).

In this study, we have found that a more precise and careful classification, using not only the classic risk factors for MS but also kidney function, is capable of identifying subtypes of patients with greater cardiovascular risk, characterized by worse blood pressure behaviors. As regards this, masked hypertension (normal office BP associated with high ambulatory BP values) seems to be more common in overweight (17.1%) or obese (30.9%) patients, compared with the non-obese patients (5.7%) (34). These data suggest that in obese subjects greater attention must be paid to early and accurate diagnosis of hypertension.

In addition to greater blood pressure load, the more advanced CKM subtypes are also characterized by greater kidney damage, expressed as increased 24-h urinary protein excretion, as well as cardiac and vascular remodeling, characterized as ventricular hypertrophy, reduced ejection fraction, larger aortic bulb diameter, and altered microvascular circulation.

Several studies confirm that obesity is strictly and independently associated with cardiac remodeling (35), especially increased LV cavity size, wall thickness, and left ventricular hypertrophy (LVH), with a predominance of eccentric cardiac hypertrophy (35). These changes concerning both structural and functional alterations are secondary to several mechanisms, as well as visceral fat distribution and related peptides secreted from epicardial adipose tissue (adipokines, angiotensin II, inflammatory cytokines); hyperinsulinemia and insulin resistance; elevated blood pressure behavior; and sleep apnea with its effect on nocturnal blood pressure, adrenergic stimulation, and chronic hypoxemia (36). In a review of data obtained from the Framingham Heart Study, obesity was independently associated with an approximate twofold higher risk for the development of HF (37). Furthermore, in epidemiological studies Khan et al. have shown a close relationship between adiposity and HF risk; in particular in older adults (>60 years), the cumulative incidence of HF for normal weight, overweight, obese patients was 9%, 13.7%, and 25.3%, respectively (38).

The individual components of MS can influence the progression of chronic nephropathy and CKD. In particular, in obese patients with CKD the renal function loss can be faster, with severe histological damage, and higher proteinuria (39). Nonetheless, as these risk factors are all potentially reversible, this offers a clear opportunity to improve CKD treatment and prevention in clinical practice (39).

Obesity and visceral adiposity can impair kidney function both directly on the kidney (called “obesity-related glomerulopathy”) and indirectly due to systemic complications that characterize obesity, such as diabetes mellitus, hypertension, and atherosclerosis (40). As regards this, the pathophysiological mechanisms of worsening of renal function are related to the different mechanisms concerning chronic low-grade inflammation, oxidative stress, endothelial dysfunction, upregulation of the renin angiotensin aldosterone system (RAAS) system, increased sympathetic nervous system (SNS) activity, and altered imbalance of adipokines (41, 42). The obesity-related glomerulopathy is characterized by greater renal hemodynamic and metabolic demand causing hyperfiltration, due to vasodilatation of the afferent arteriole, increased salt reabsorption in proximal tubular, greater glomerular tuft volume, and later glomerulosclerosis (43). Conversely, hyperfiltration is a strong contributor to development of renal damage favoring hypertension in humans (44). Kidney histopathological alterations can be characterized by glomerulomegaly alone or with secondary focal segmental glomerulosclerosis in the glomerular perihilar area, decreased podocyte density, increased mesangial matrix, and mesangial sclerosis (45–47); all these alterations manifest themselves not only with a reduction in glomerular filtration but also as an increase in urinary protein excretion (48).

Obesity is a serious public health issue, well recognized for being associated with an increased CVD risk, directly related to excess body fat mass, as well as other obesity-related complications such as pre-type 2 diabetes, obstructive sleep apnea, and non-alcoholic fatty liver diseases. Diet and physical activity are key points in preventing CV disease and reducing CV risk; however, these strategies alone are not always sufficient. A multidisciplinary approach, complex pharmacology, and bariatric surgery are effective in reducing the incidence of death and cardiovascular events (49, 50).

Regarding pharmacological treatment, for several decades it has been well documented that RAAS blockers, particularly in the presence of albuminuria or proteinuria, should be administered to reduce not only arterial hypertension and the increased activation of the system but also sympathetic overactivity, insulin resistance, and low-grade inflammation, with the indication of close control of renal function indices (42). Recently, beyond improvement in both weight and glycemia control, new anti-diabetic drugs, such as glucagon-like peptide 1 receptor agonists (GLP-1RA) and sodium-dependent glucose co-transporter proteins 1 and 2 (SGLT1/2), were shown to be useful in reducing cardiovascular and kidney outcomes (death from cardiovascular disease, non-fatal myocardial infarction, non-fatal cerebral infarction, progressive decline in the eGFR of at least 50%, or end-stage kidney disease) in patients with diabetes with and without CKD (51–53). Thus, International Consensus has supported the preferred use of GLP-1 RAs and SGLT2i in the treatment of patients with type 2 diabetes mellitus (T2DM) and CKD (54).

In conclusion, visceral obesity and MS are a frequent condition with high impact on health status, becoming important triggers for the development of hypertension and cardiovascular and metabolic complications. The different classifications of MS allow the early identification of those subjects at high risk of developing cardiometabolic complications. Recently identified, the CKM syndrome is a dynamic assessment of MS compared with previous classifications with a more prevalent cardiorenal phenotype that is obesity related. The new CKM syndrome, characterized by different stages, proves useful to better identify individuals at high risk for CKM morbidity and mortality, to start early treatment before onset or worsening of organ damage.

## Data Availability

The raw data supporting the conclusions of this article will be made available by the authors, without undue reservation.
